# Enhanced Adsorption of Textile Dyes by a Novel Sulfonated Activated Carbon Derived from Pomegranate Peel Waste: Isotherm, Kinetic and Thermodynamic Study

**DOI:** 10.3390/molecules28237712

**Published:** 2023-11-22

**Authors:** Badr M. Thamer, Faiz A. Al-aizari, Hany S. Abdo

**Affiliations:** 1 Department of Chemistry, College of Science, King Saud University, Riyadh 11451, Saudi Arabia; faizalaizari@yahoo.com; 2 Mechanical Engineering Department, College of Engineering, King Saud University, Riyadh 11421, Saudi Arabia; habdo@ksu.edu.sa

**Keywords:** activated carbon, surface modification, sulfonated activated carbon, adsorption, crystal violet dye, isotherm, kinetics

## Abstract

The rapid growth of the dye and textile industry has raised significant public concerns regarding the pollution caused by dye wastewater, which poses potential risks to human health. In this study, we successfully improved the adsorption efficiency of activated carbon derived from pomegranate peel waste (PPAC) through a single-step and surface modification approach using 5-sulfonate-salicylaldehyde sodium salt. This innovative and effective sulfonation approach to produce sulfonated activated carbon (S-PPAC) proved to be highly effective in removing crystal violet dye (CV) from polluted water. The prepared PPAC and S-PPAC were characterized via FESEM, EDS, FTIR and BET surface area. Characterization studies confirmed the highly porous structure of the PPAC and its successful surface modification, with surface areas reaching 1180.63 m^2^/g and 740.75 m^2^/g for the PPAC and S-PPAC, respectively. The maximum adsorption capacity was achieved at 785.53 mg/g with the S-PPAC, an increase of 22.76% compared to the PPAC at 45 °C. The isothermic adsorption and kinetic studies demonstrated that the adsorption process aligned well with the Freundlich isotherm model and followed the Elovich kinetic model, respectively. The thermodynamic study confirmed that the adsorption of CV dye was endothermic, spontaneous and thermodynamically favorable onto PPAC and S-PPAC.

## 1. Introduction

Water pollution is one of the environmental problems facing many industrialized countries. Therefore, reusing industrial wastewater is an environmental necessity to meet the acute shortage of groundwater needed for agricultural and other activities, and to limit its leakage into ecosystems [1,2]. The discharge of untreated or partially treated dyed wastewater from textile industries, ink, cosmetics and medical laboratories into water bodies is a major source of water pollution [3,4]. The leakage of dyes into aquatic ecosystems causes some problems, including decreased oxygen levels in bodies of water and less sunlight penetration, which impedes photosynthesis and further harms the ecology [5,6,7]. Furthermore, many dyes are poisonous compounds that endanger aquatic organisms. Several techniques for removing colors from wastewater have been developed to address the negative environmental implications of dyed wastewater [8]. Sedimentation, filtration and membrane processes are examples of physical methods, while coagulation, flocculation and oxidation are examples of chemical methods [9]. While these strategies have proven to be somewhat effective, adsorption technology has emerged as a particularly essential option for dyes removal over the past decades [10]. Adsorption is a versatile and cost-effective process that involves the attachment of adsorbate molecules to a solid adsorbent, for the contaminants removal from wastewater [11]. Thus, this method has gained attention in wastewater treatment due to its adaptability and effectiveness across a broad spectrum of dyes. The advantages of adsorption processes include their simplicity of implementation, high efficiency in dye removal, and the potential for regeneration of the adsorbent, enabling its reuse [12,13]. This technique is particularly valuable for treating wastewater containing persistent and challenging-to-treat dyes, as it provides a viable solution where other methods may be inefficient [14]. Several materials have been found to be effective in adsorbing dyes from wastewater treatment. Activated carbon can be made from a variety of biowaste products and used as an adsorbent for dyes in wastewater treatment, providing both environmental and economic benefits [15]. Agricultural waste, coconut shells, sawdust and fruit peels can all be used as precursors for activated carbon production [16]. The carbonization of biowaste followed by activation, either physical or chemical, is one of the most common processes used to prepare activated carbon [17]. The activated carbon has distinct properties that make it extremely effective for the adsorption various dyes and other contaminants. Its porous structure, derived from the original biowaste material, has a wide surface area and an abundance of pore volume, allowing for efficient dyes adsorption [18,19]. As a result of its chemical composition and surface chemistry, activated carbon also has significant adsorption capacity, allowing for numerous interactions such as hydrogen bonding, π–π stacking and electrostatic interactions with dyes [20]. Furthermore, biowaste-derived activated carbon has the advantage of utilizing renewable and sustainable resources, minimizing waste output and contributing to a circular economy [21]. Surface functionalization, a method that includes altering the surface chemistry of activated carbon, can improves its adsorption capacity for dyes removal even further [22]. Functionalization of activated carbon can be achieved through chemical modification of its surface or by incorporating external materials. This change enhances the availability of active adsorption sites accessible and introduces particular interactions between functional groups and dye molecules. The grafting of hydroxyl, carboxyl or amine groups, for example, allows for increased hydrogen bonding, electrostatic interactions and π–π stacking interactions with dyes, hence increasing the adsorption capacity [23]. Surface-functionalized activated carbon is a highly efficient adsorbent for effective contaminants removal from wastewater due to its customized surface characteristics and improved affinity towards dye molecules. For example, Iannicelli-Zubiani et al. enhanced the adsorption efficiency of activated carbon for lanthanum ions removal by pentaethylenehexamine [24]. The results demonstrated the functionalized activated carbon maintained its adsorption performance after four reuse cycles. Goswami and Phukan et al. modified activated carbon with sulfonic acid and found that the functionalized activated carbon has a maximum adsorption capacity that is higher than that of pristine activated carbon for the adsorption of cationic dyes [25]. To our knowledge, there has been no research that explored the surface modification of activated carbon by 5-sulfonato-salicylaldehyde sodium salt.

In this study, activated carbon was synthesized using a one-step carbonization/activation process of pomegranate peels followed a simple, novel and successful sulfonation process for improved dye adsorption. The sulfonation process was carried out by covalent bonding between activated carbon and 5-sulfonato-salicylaldehyde sodium salt. Sodium sulfonate groups (-SO_3_^−^Na^+^) provide a negative charge on the PPAC surface, making it a potential candidate for the adsorption of cationic dye molecules. Various techniques were used to characterize the synthesized PPAC, including Fourier transform infrared spectroscopy (FTIR), scanning electron microscopy (SEM), and Brunauer–Emmett–Teller (BET) surface area analysis. The existence of -SO_3_^−^Na^+^ groups on the surface of the AC was confirmed via the FTIR data and its surface charge nature was investigated with a drift method. SEM images revealed that the AC has a porous structure, which is advantageous for adsorption. The BET surface areas of the activated carbon (PPAC) and sulfonated activated carbon (S-PPAC) were determined to be 1180.63 and 740.75 m^2^/g, respectively. A preliminary study was carried out to explore the adsorption efficiency of PPAC and S-PPAC towards cationic and anionic dyes. PPAC and S-PPAC are highly efficient adsorbents for a wide range of dyes, but they are especially effective for cationic crystal violet dye.

## 2. Results

### 2.1. Characterization

**Figure 1:**
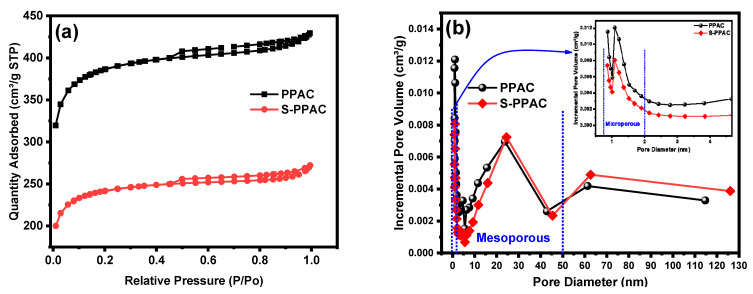
(**a**) N_2_ adsorption–desorption isotherms and (**b**) the pore size distributions of PPAC and S-PPAC.

The physical properties (e.g., surface area, porosity) and surface properties (e.g., nature functional groups) of the adsorbent and its morphology are among the most important factors that play a prominent role in determining its adsorption efficiency against various pollutants. The N_2_ adsorption and desorption isotherms were used to investigate the microstructures of PPAC and S-PPAC (Figure 1a). PPAC exhibited a typical type I isotherm with an H_4_ hysteresis loop, and its adsorption capacity increased significantly at low initial relative pressures, which is consistent with the isotherm properties of microporous materials [26]. These findings indicate that the pore structure of PPAC is largely microporous, which is consistent with the pore size distribution curve (insert Figure 1b). The high surface area and porosity of PPAC and S-PPAC were validated using BET analysis, and the results are summarized in Table 1. PPAC has a specific surface area (S_BET_) of 1180.63 m^2^/g, a total pore volume (V_T_) of 0.6644 cm^3^/g and a microporous surface area of 75.45% (Section S1). S-PPAC, on the other hand, showed decreases in the specific surface area (740.75 m^2^/g) and total pore volume (0.421 cm^3^/g) after the sulfonation process. This can be attributed to clogged pores. Table 1 lists the textural properties of the PPAC and S-PPAC samples. Chemical modification of activated carbon can often lead to a decrease in surface area. This is because the chemical modification process can block pores or collapse of pore walls, which aligns with numerous studies that have observed similar outcomes [27,28,29]. Although chemical modification processes lead to reduce surface areas, the modified activated carbon exhibits a significant presence of polar functional groups. This advantageous feature renders the activated carbon surface more hydrophilic and facilitates interaction with ionic pollutants. To confirm the hydrophilic nature, the water absorption ratios on the PPAC and S-PPAC surfaces were calculated, where the absorption ratio for S-PPAC reached 820%, while it reached 430% for unmodified PPAC. These results confirm the successful functionalization process and hydrophilic nature of the S-PPAC.

**Figure 2:**
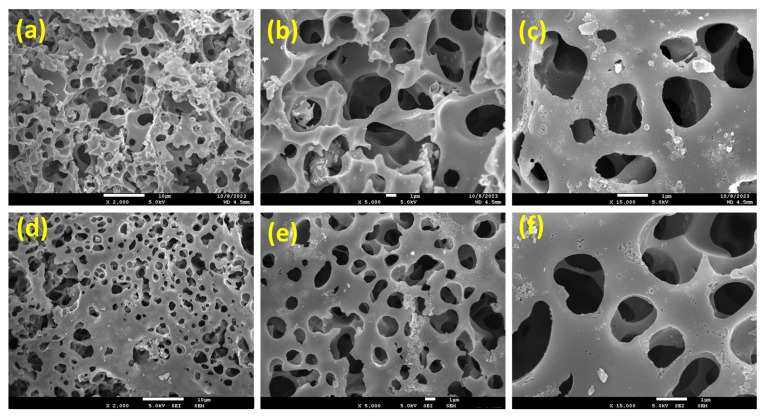
FESEM images of (**a**–**c**) PPAC and (**d**–**f**) S-PPAC at 2000× (scale bar 10 μm), 5000× (scale bar 1 μm) and 15,000× (scale bar 1 μm) magnifications.

The morphological characteristics of PPAC and S-PPAC were investigated using FESEM. Figure 2a–c depict the surface morphologies of the PPAC at different magnifications, which appeared as honeycomb-like pores with irregular and heterogeneous surface morphologies with a well-developed porous structure. However, upon treatment with a sulfonation agent, the PPAC surface became less rough, with a slight decrease in pore size (Figure 2d–f).

**Figure 3:**
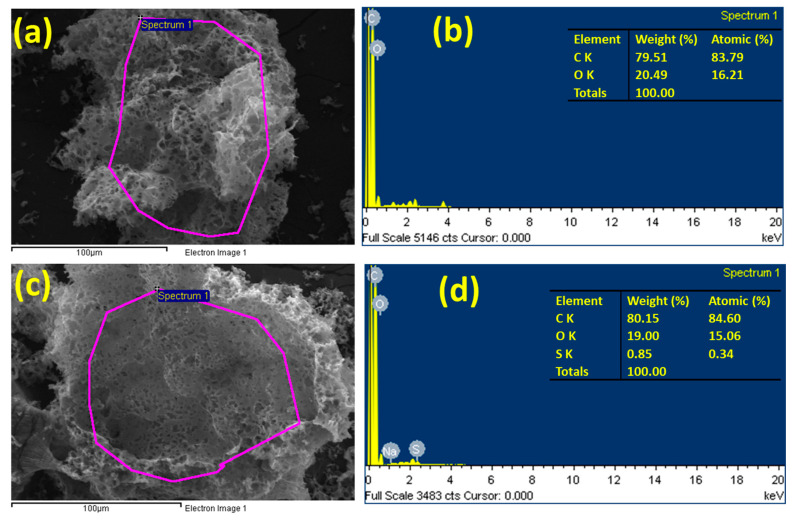
EDS analysis of (**a**,**b**) PPAC and (**c**,**d**) S-PPAC.

Quantitative energy dispersive X-ray spectroscopy (EDS) was used to examine the elemental composition and conformation of the -SO_3_H groups on the S-PPAC. Figure 3 depicts the results, which show the presence of sulfur in the S-PPAC. The quantitative analysis determines the carbon, oxygen and sulfur element mass ratios in the sulfonated PPAC to be 80.15%, 19% and 0.85%, respectively.

**Figure 4:**
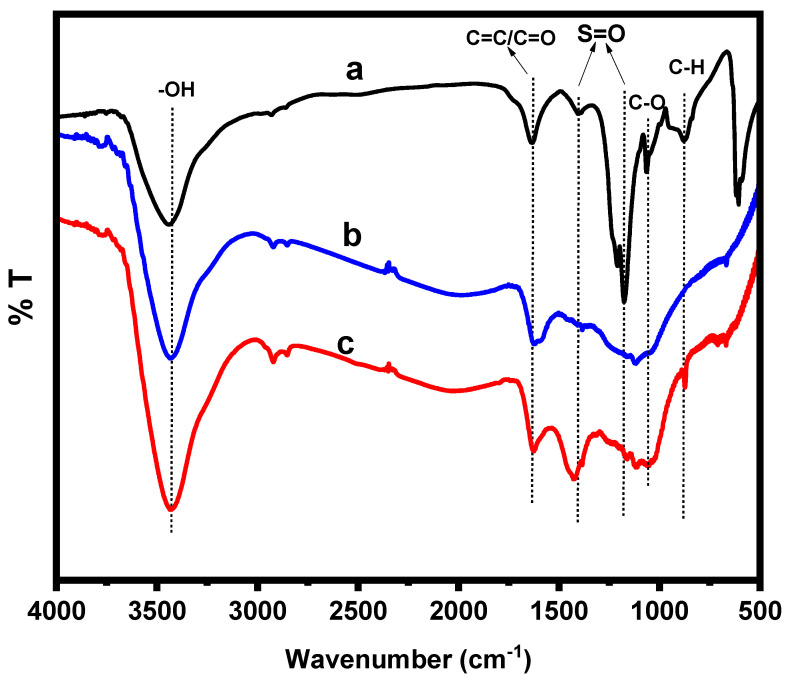
FT-IR spectra of (**a**) 5-SSADS agent, (**b**) PPAC and (**c**) S-PPAC.

The functional groups attached on the PPAC surface before and after the sulfonation process were identified using FTIR analysis. The FTIR spectrum of the prepared 5-SSADS agent, as shown in Figure 4a, displays distinctive absorption bands at 3441, 1630, 1400 and 1173 cm^−1^, corresponding to the O-H stretching vibrations, C=O/C=C stretching vibrations and S=O stretching vibrations, respectively. The spectrum also has two peaks at 1206 and 1062 cm^−1^, which are attributed to asymmetric and symmetric stretching of C–O, while the peak at 876 cm^−1^ is attributed to C-H of 1,2,4 trisubstitution. The FTIR spectrum of PPAC (Figure 4b) reveals a peak at 3437 cm^−1^ corresponding to the bonded –OH group. The peaks at 1625, 1597 and 1383 cm^−1^ could be assigned to C=O stretching vibrations of the carboxylic group. The peaks at 1160 and 1119 cm^−1^ could be attributed to C–O stretching vibrations of carboxylic acid, phenols, alcohols or esters [30]. Upon sulfonation with 5-SSADS, new bands emerged at 1425 and 874 cm^−1^, confirming the presence of sulfonate groups and C-H of 1,2,4 trisubstitution, which validate a successful sulfonation process (Figure 4c).

### 2.2. Adsorption Study

#### 2.2.1. Test Adsorption of Various Dyes

**Figure 5:**
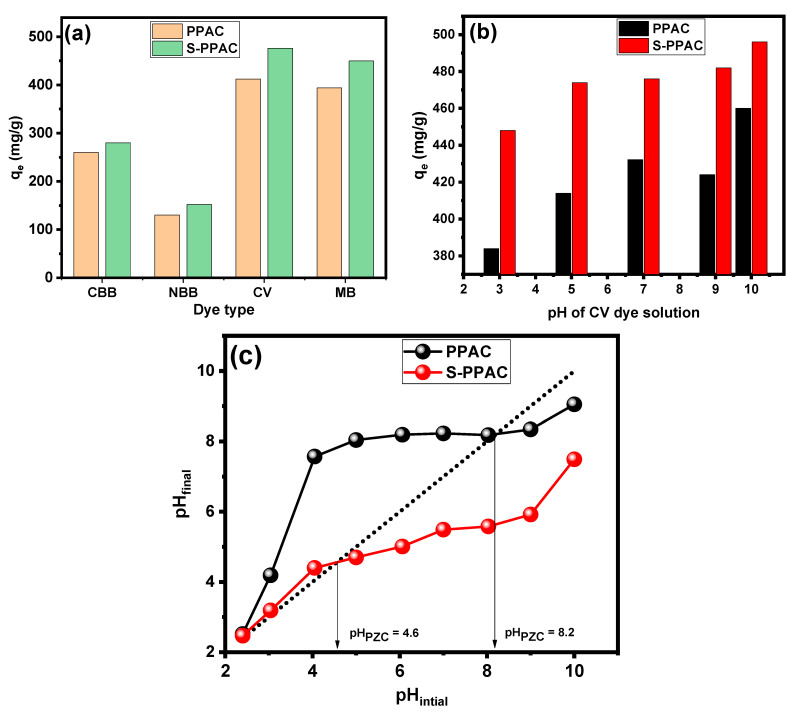
(**a**) Test adsorption capacities of PPAC and S-PPAC for various cationic and anionic dyes, (**b**) effect of pH on adsorption of CV dye and (**c**) plot of initial pH vs. final pH to estimate zero-point charge (pH_ZPC_) values for PPAC and S-PPAC.

A preliminary adsorption survey was performed to identify the most adsorbable organic dyes, both cationic and anionic, on the surfaces of the PPAC and S-PPAC. Four dyes were selected, including two cationic dyes (crystal violet (CV) and methylene blue (MB)) and two anionic dyes (Coomassie brilliant blue (CBB) and naphthol blue black (NBB)). The test was performed without any pH adjustment and at concentrations of 300 mg/L for each dye, as shown in Figure 5a. The results showed that cationic dyes have higher absorbency than anionic dyes on the surfaces of both adsorbents. This is likely due to electrostatic attraction between the positively charged dyes and the negatively charged surfaces of the PPAC and S-PPAC. The results also show that the modified activated carbon (S-PPAC) has a higher adsorption capacity than the unmodified activated carbon towards various dyes. It was also observed that the cationic CV dye exhibited higher adsorption capacity than the cationic MB dye. Based on these findings, CV dye was chosen for further investigation into the parameters influencing the adsorption process. The pH, contact time, initial dye concentration and temperature were all investigated.

#### 2.2.2. Effect of pH on Adsorption of CV Dye

Several studies highlighted that the initial pH of a solution is a crucial environmental factor that influences the adsorption process. It impacts various aspects such as the adsorbate solubility and ionization degree as well as the surface charge of the adsorbent [31,32,33]. In this study, the effects of initial pH on the adsorption capacities of PPAC and S-PPAC for the removal of CV dye were investigated within a pH range of 3.0 to 10.0. Figure 5b presents the influence of initial pH on dye removal, while keeping constant the initial dye concentration (300 mg/L), contact time (24 h), temperature (25 °C) and adsorbent dosage (0.5 g/L). The adsorption capacity of PPAC increased with the pH increasing from 3 to 7. However, a further increase in the pH above 7 resulted in a slight decrease in adsorption capacity. It was observed that PPAC exhibited favorable adsorption, achieving a maximum uptake of CV dye (466 mg/g) at pH 7.0. The relatively minor reductions in the adsorption capacity of PPAC at conditions of higher pH_ZPC_ can be due to molecules forming zwitterions/dimerization of the CV dye molecules, making diffusion into the pores difficult. Moreover, it can be said that the high adsorption capacity of PPAC under a pH of less than the pH_ZPC_ value confirms that there are factors other than electrostatic attraction that play a prominent role in the adsorption of the CV dye on the PPAC, such as hydrogen bonding and filling of pores. On the other hand, the S-PPAC demonstrated a maximum uptake of CV dye with a value of 496 mg/g at pH 10.0. The values of the zero-point charge (pH_ZPC_) for PPAC and S-PPAC were determined to be 8.2 and 4.6, respectively, as shown in Figure 5c. For pH values above the pH_ZPC_, the deprotonation of surface sites on S-PPAC increases the number of negatively charged sites, attracting the positively charged cationic CV dye. Although the surface area of the S-PPAC is less than that of the unmodified PPAC, its adsorption performance is higher, which indicates that surface functional groups such as sulfonates play a main role in enhancing electrostatic attraction with the CV dye onto the S-PPAC surface. Also, even at pH 3.0, both the PPAC and S-PPAC displayed a significant capability for adsorbing CV dye. This finding is consistent with reported studies on CV adsorption onto charcoal [34] and activated carbon [35]. These findings imply the presence of other substantial contributions, such as pore-filling, hydrogen bonding and π–π interactions, all of which play an important role in the adsorption process.

#### 2.2.3. Effect of Initial CV Dye Concentration and Isotherm Study

**Figure 6:**
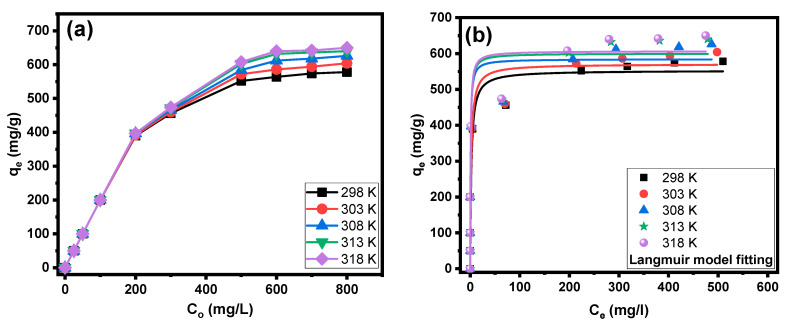

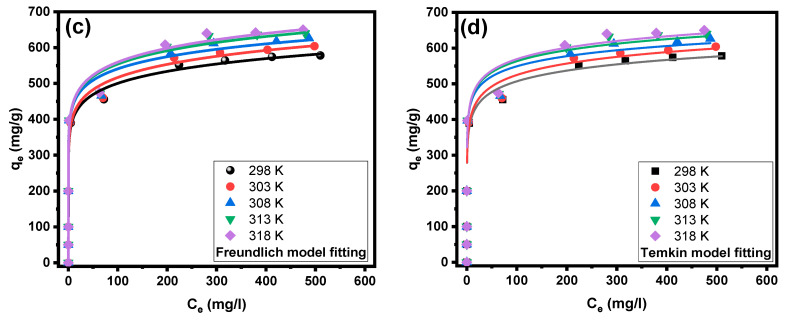
(**a**) Initial concentration effects at various temperatures on adsorption of CV dye onto PPAC; (**b**) Langmuir fitting, (**c**) Freundlich fitting and (**d**) Temkin fitting.

**Figure 7:**
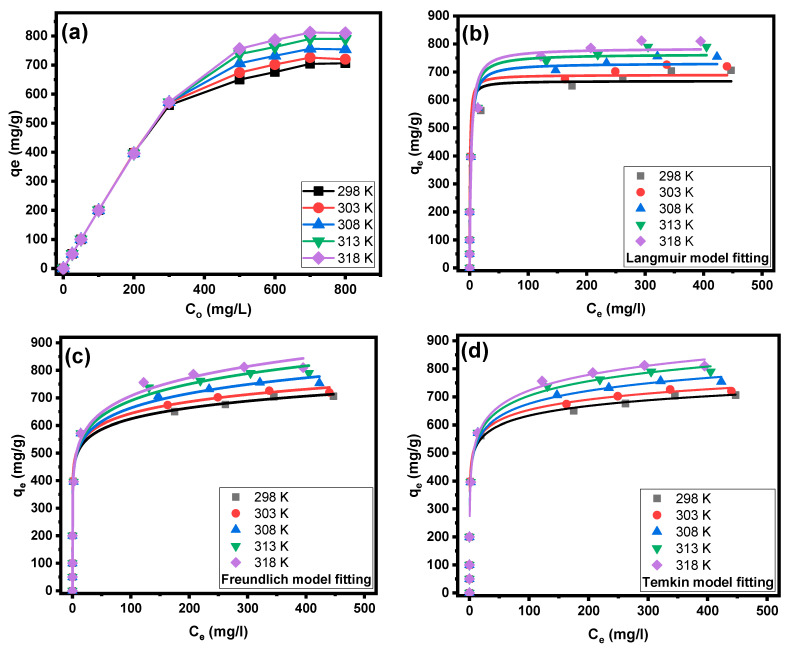
(**a**) Initial concentration effects at various temperatures on adsorption of CV dye onto S-PPAC; (**b**) Langmuir fitting, (**c**) Freundlich fitting and (**d**) Temkin fitting.

Figure 6a and Figure 7a illustrate the impacts of the initial concentration and solution temperature of the CV dye on its sorption by PPAC and S-PPAC. At different temperatures, the adsorption capacities of PPAC and S-PPAC increase linearly in concentration ranges of 25 to 200 mg/L and 25 to 300 mg/L, respectively. This shows that the CV dye was totally eliminated at this concentration range. At concentrations higher than 200 mg/L for PPAC and 300 mg/L for S-PPAC, the adsorption capacity continues to increase with increasing concentration and temperature. The observed behaviors can be explained by the presence of a significant driving force during the adsorption process, which is characterized by high initial concentrations. The saturation adsorption of CV dye onto PPAC approaches a condition of near-constancy at 600 mg/L, while S-PPAC reaches equilibrium at 700 mg/L. This shows that S-PPAC has more adsorption sites on its surface than PPAC. At a concentration of 800 mg/L for the CV dye, the maximum adsorption capacities are 578, 604, 626, 640 and 650 at 298, 303, 308, 313 and 318 K, respectively, for PPAC. In contrast, the adsorption capacities are 706, 720, 754, 790 and 810 mg/g at 298, 303, 308, 313 and 318 K, respectively, for PPAC. This behavior indicates that the adsorption process is an endothermic process, and its increase facilitates the penetration of the dye into the pores of PPAC and S-PPAC.

Three adsorption nonlinear isotherm models were used to investigate the equilibrium adsorption behavior: Langmuir, Freundlich and Temkin. Figure 6b–d and Figure 7b–d show the Langmuir, Freundlich and Temkin isotherm plots for the adsorption of CV dye onto PPAC and S-PPAC, respectively, at various temperatures. Table 2 lists the characteristic parameters of those models at various temperatures, as well as the correlation coefficients (R^2^). The obtained results show good agreement between the experimentally acquired data and the Freundlich model, surpassing other models in terms of the fits shown in Figure 6b and Figure 7b. This observation can be attributed to the much higher correlation coefficient (R^2^) value obtained for the Freundlich isotherm, with values between 0.8923 and 0.9094 for PPAC and 0.9299 and 0.9456 for S-PPAC. Fitting the experimental data using the Freundlich model indicates that the adsorption is multilayered on the surfaces of the PPAC and S-PPAC and on a heterogeneous surface. The parameter “1/n” has been used to represent the degree of favorability associated with this particular form of adsorption. A value of 1/n less than 0.5 indicates that the adsorbate is easily adsorbed, whereas a value of 1/n more than 2 indicates that the adsorbate is little adsorbed [36]. This indicates that the adsorption of CV dye onto PPAC and S-PPAC, as described by the Freundlich model, can be considered favorable. Due to the inability of the Freundlich model to calculate the adsorption capacity, it can be calculated using the Langmuir model, as the R^2^ values of the Langmuir model closely align with those of the Freundlich model. Notably, the adsorption capacity of S-PPAC at different temperatures exceeds that of PPAC, indicating the efficacy of the sulfonation process in enhancing adsorption sites through the introduction of sulfonate groups. Specifically, the adsorption capacity value of PPAC is 552.79 mg/g at 298 K and increases to 606.7 mg/g as the temperature rises to 318 K. Similarly, the adsorption capacity of S-PPAC increases from 667.84 to 785.53 m/g as the temperature elevates from 298 to 318 K.

Table 3 presents a comprehensive comparison of S-PPAC with various adsorbents previously employed for the removal of CV dyes investigated in this study [34,37,38,39,40,41,42,43,44,45,46,47]. PPAC has a higher surface area (1180.63 m^2^/g) than other types of activated carbon reported in the literature [34,40,45]. It is evident from Table 2 that the adsorption capacities of S-PPAC are higher than the reported adsorbents, although some adsorbents exhibited higher surface areas [38,44,46,47]. These findings suggest that S-PPAC holds promise as an effective adsorbent for purifying wastewater contaminated with cationic dyes.

#### 2.2.4. Effect of Contact Time and Kinetic Study

**Figure 8:**
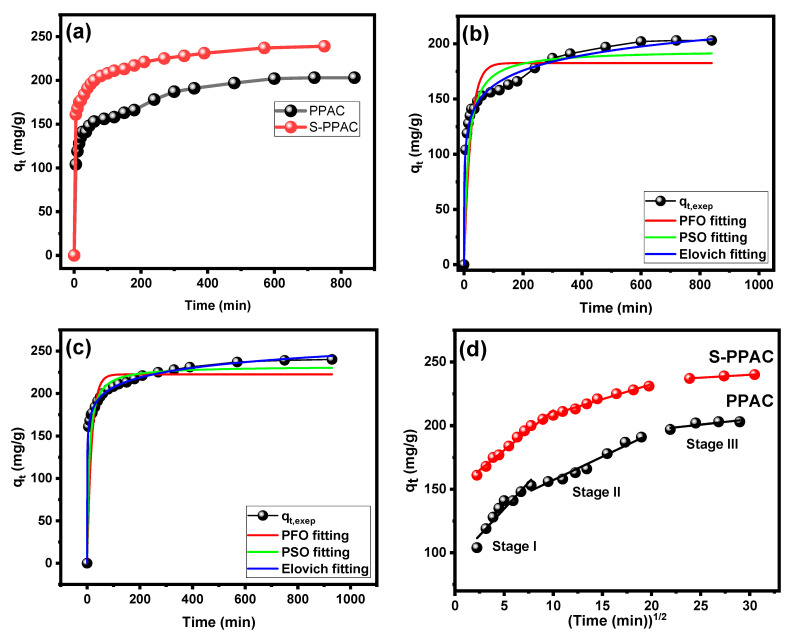
(**a**) Contact adsorption time effect on CV dye adsorption; kinetic model fittings for adsorption data of (**b**) PPAC and (**c**) S-PPAC; and (**d**) intraparticle diffusion model.

The contact time plays a crucial role in the adsorption process, and its optimization was performed within the range of 5–900 min for a 300 mg/L concentration of CV dye. The obtained results (Figure 8a) indicate that the efficiencies of dye removal exceeded 34.66% (104 mg/g) and 53.66% (161 mg/g) during the initial 5 min by PPAC and S-PPAC, respectively. This rapid adsorption of CV dye molecules onto S-PPAC can be attributed to the abundance of vacant sites on its surface, which are readily occupied upon exposure to the CV dye molecules. Although the initial minutes show a high rate of adsorption onto the surfaces of both the PPAC and S-PPAC, the adsorption process continues beyond this point and gradually approaches equilibrium. This equilibrium state is attained after 300 min for PPAC and around 210 min for S-PPAC. The high adsorption equilibrium times can be due to the existence of large interfacial pores on the PPAC and S-PPAC surfaces, which require additional time for the CV dye molecules to penetrate and occupy these spaces.

To acquire a better understanding of the adsorption behavior of PPAC and S-PPAC towards CV dye, the adsorption kinetic data were analyzed using three commonly used non-linear kinetics models: pseudo-first-order (PFO), pseudo-second-order (PSO) and Elovich. The Supplementary Information (SI) contains detailed explanations of these three models. The non-linear curve produced from the Elovich model provides the best fitting results for CV dye adsorption onto PPAC and S-PPAC, as shown in Figure 8c. Table 4 shows that the correlation coefficient R^2^ values of the Elovich model exceed 0.999 for PPAC and S-PPAC. Furthermore, the Elovich model has the lowest chi-square (χ^2^) values, measuring 16.27 and 2.66, whereas the PFO has 228.8 and 133.62 and the PSO has 105.40 and 45.36 for the PPAC and S-PPAC, respectively.

In order to gain a deeper understanding of the adsorption process of PPAC and S-PPAC towards CV dye, the intraparticle diffusion model was utilized to analyze the rate-controlling step of adsorption. The equation for this model is as follows:

$$q_{t}=K_{p}t^{0.5}+C$$

Here, C and k_p_ (mg/g min^0.5^) represent the boundary layer diffusion effect and the intraparticle diffusion rate constant, respectively.

Figure 8d depicts the intraparticle diffusion curves. The adsorption of PPAC and S-PPAC towards CV dye produced multilinear plots, showing that three stages are involved in the adsorption process. The diffusion rate is highest in the first linear segment, indicating that external diffusion is the rate-limiting step. This is due to CV dye diffusion from the solution to the boundary layer surrounding the adsorbent or across the boundary layer to the external surface. The diffusion rate in the second linear portion decreases, which can be attributed to the diffusion of CV dye from the exterior surface to the internal pores of the PPAC and S-PPAC. Thus, in this second segment, pore diffusion or intraparticle diffusion becomes the rate-controlling step. During the final equilibrium stage, the third portion exhibits a low diffusion rate. According to the three-stage mechanism, the adsorption of CV dye onto PPAC and S-PPAC involves both intraparticle and exterior diffusion.

#### 2.2.5. Thermodynamic Study and Proposed Adsorption Mechanism

Based on the results of the effect of temperature on the adsorption of CV dye on the surface of PPAC and as discussed in Section 2.2.3, the results show an increase in the adsorption capacity with increasing temperature in the temperature range from 298 to 318 K, which indicates that the adsorption of the dye onto both adsorbents was endothermic. Temperature plays a dual role in influencing the adsorption process. Firstly, it reduces the solution viscosity, which facilitates the movement of adsorbate molecules across the adsorbent’s external boundary layer and within its internal pores. This acceleration of adsorbate diffusion enhances the overall adsorption rate, leading to more efficient removal of contaminants from the solution. Secondly, temperature changes affect the adsorbent’s equilibrium capacity for a certain adsorbate [48]. The increased temperature promotes disaggregation, which aids in the uptake of CV molecule monomers.

**Figure 9:**
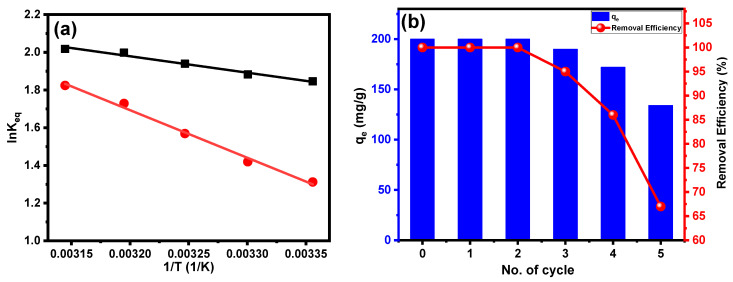
(**a**) Plot of lnKeq versus 1/T for estimating the thermodynamic parameters associated with the adsorption of CV dye onto PPAC and S-PPAC and (**b**) reusability of S-PPAC for adsorption CV dye.

To acquire a better understanding of the adsorption mechanism, several thermodynamic parameters for the system were determined, such as the adsorption free energy (ΔG°), the adsorption enthalpy (ΔH°) and the adsorption entropy (ΔS°). The van’t Hoff plot’s slope and intercept correspond to ΔH°/R and ΔS°/R, respectively, where R represents the universal gas constant (8.314 J/(mol K)) and T signifies the absolute temperature in Kelvin, as shown in Figure 9a. The following equation is used to calculate the change in ΔG° of adsorption:

∆G°=−RTlnKc

$$lnK_{c}=\frac{∆S{}^{\circ}}{R}-\frac{∆H{}^{\circ}}{RT}$$

where R represents the gas constant, K represents the equilibrium constant and T represents the temperature in Kelvin in this equation. Table 5 summarizes the obtained thermodynamic parameters.

Notably, all of the ΔG° values in Table 5 are negative, showing a spontaneous adsorption process with a substantial preference for CV dye on PPAC and S-PPAC. The ΔG° values for the adsorption of CV dye onto PPAC and S-PPAC exhibit variations within a range, with mean values gradually increasing from −4.57 to −5.34 kJ/mol and from −3.25 to −4.82 KJ/mol for temperatures increasing from 293 to 318 K, respectively. In addition, as shown in Table 5, the positive ΔH° value indicates that the adsorption process is endothermic. The hydration extent of cationic dye molecules and the disaggregation of dimers or aggregates generated during the process are responsible for the positive ΔS°. Similarly, positive values of enthalpy and entropy have been reported in the literature for the adsorption of CV dye in solution onto activated carbon, such as chitin-derived activated carbon (ΔH° = 38.95 kJ/mol and ΔS°= 136.79 J/mol, respectively) [38], Moroccan Moringa oleifera waste-derived activated carbon (ΔH° = 17.02 kJ/mol and ΔS° = 64 J/mol, respectively) [46] and activated carbon derived from coconut palm male flowers (ΔH° = 71.49 kJ/mol and ΔS°= 258.67 J/mol, respectively) [49]. Positive changes in entropy can result from a variety of sources, including increased disorder of the solvent molecules in the surrounding solution or higher mobility and freedom of the adsorbate molecules upon attachment to the activated canters on the surface. This greater disorder leads to a positive ΔS°, which can favor the spontaneity of the activity.

#### 2.2.6. Reusability Study

Reusing adsorbents provides a valuable solution to reduce the overall cost of the adsorption process, since the adsorbent is the core of the process. This approach not only contributes to cost savings, but also enhances sustainability by reducing waste generation and resource consumption, and represents an effective strategy to enhance the economic viability and environmental impact of adsorption processes. In this study, the potential for reusing S-PPAC for CV dye adsorption was investigated under the following experimental conditions: a CV dye concentration of 100 mg/L, an S-PPAC dose of 0.5 g/L and an adsorption time of 4 h. For desorption, a mixture of HCl (0.1 M) and acetone was used in a ratio of 75:25. The results of the reuse (Figure 9b) experiment demonstrate that S-PPAC can be successfully reused for three consecutive cycles without any noticeable loss in the adsorption efficiency. However, in the fourth cycle, the efficiency decreases to 86%. Subsequently, in the fifth cycle, the efficiency further declines to 67%. These findings suggest that the S-PPAC has the potential for three reuse cycles while retaining its adsorption efficiency. However, following the third cycle, there was a progressive decline in efficiency, with a more significant loss noted in the fourth and fifth cycles.

## 3. Materials and Methods

### 3.1. Materials

Salicylic benzaldehyde, sulfuric acid, sodium carbonate, sodium hydroxide, acetone, acetic acid, hydrochloric acid, potassium hydroxide and crystal violet dye were purchased from Sigma-Aldrich Co., Steinheim, Germany.

### 3.2. Preparation of Activated Carbon and Sulfonated Activated Carbon

The used pomegranate peel (PP) in this study was sourced from Yemen and subjected to a drying process in an environment devoid of sunlight for several weeks. Following this, the PP was subjected to two rounds of washing with distilled water. Subsequently, the dried PP was placed in a vacuum oven and dried at a temperature of 80 °C. After drying, the PP was grinded using a blender to obtain fine powder. To prepare the PP/KOH mixture, 10 g of KOH was dissolved in 50 mL of distilled water. Subsequently, 10 g of the obtained PP powder was added to the KOH solution, and the mixture was stirred for a period of 3 h. Then, the mixture was poured into a ceramic crucible and subjected to a drying process at a temperature of 100 °C under vacuum. The dried mixture was then transferred to a tube furnace, and carbonized/activated at 800 °C for 2 h at a heating rate of 300 °C/h. After the carbonization/activation process was completed, the furnace was allowed to cool naturally to laboratory temperature. To remove residual salt, the black product was soaked in a sufficient amount of distilled water and stirred overnight. Finally, the product was filtered and washed multiple times with distilled water, then dried at a temperature of 100 °C in a vacuum oven; it was named activated carbon derived from pomegranate peel (PPAC).

The 5-sulfonato-salicylaldehyde sodium salt (5-SSADS) was synthesized by reacting 12.7 mL of salicylaldehyde and 125 mL of concentrated sulfuric acid in a 250 mL round bottom flask with stirring at 22 °C, and then continuous stirring for 24 h. Following that, sodium carbonate was added to the mixture that was stirred at 40 °C until a visibly identifiable pink-colored precipitate appeared. Then, the precipitate was washed with ethanol and dried in an oven vacuum at 60 °C.

**Figure 10:**
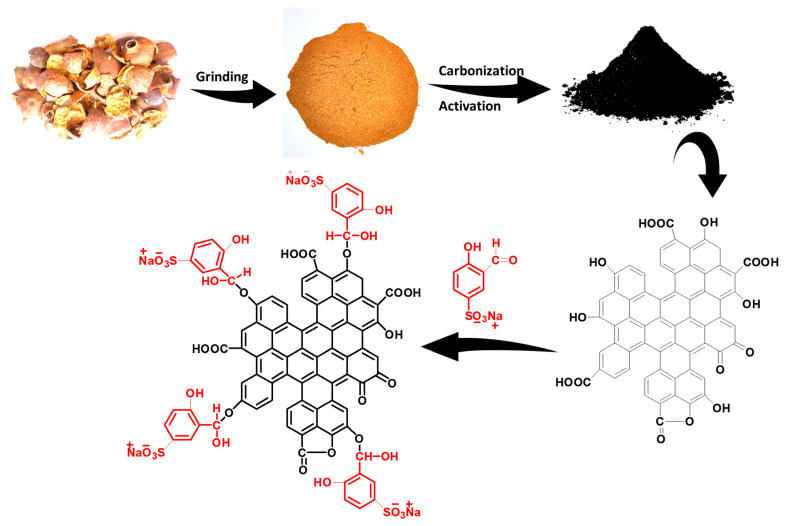
Preparation and surface modification of PPAC.

The sulfonation of PPAC was carried out by dissolving 0.2 g of 5-SSADS in 50 mL of acetic acid in a round bottom glass. Subsequently, 0.2 g of PPAC was added to the solution, which was then subjected to continuous stirring for a duration of 24 h at a temperature of 80 °C. Following this, the sulfonated activated carbon product (S-PPAC) was filtered and washed three times with distilled water to remove any residual unreacted 5-sulfonato-salicylaldehyde sodium salt. Finally, the S-PPAC was dried at 80 °C under vacuum. Figure 10 summarizes the preparation steps for the PPAC and S-PPAC.

### 3.3. Characterization

The BET surface area and pore structure features of both the PPAC and S-PPAC were measured using a N_2_ adsorption–desorption isotherm analyzer, namely the Micromeritics ASAP-2020 Autosorb-gas sorption analyzer, at −196 °C. Field emission scanning electron microscopy (FESEM, JOEL 2100, Peabody, MA, USA) was used to study the surface textures and pore morphologies of the PPAC and S-PPAC. Fourier transform infrared (FT-IR, Thermo Fisher Scientific, Waltham, MA, USA) spectroscopy was used to examine the functional groups in the samples, using a scanning range of 4000 to 400 cm^−1^.

### 3.4. Adsorption Measurements

To investigate the adsorption of dyes onto the PPAC and S-PPAC, batch adsorption experiments were conducted. A mass of 10 mg of adsorbent was added to a polypropylene tube containing 10 mL of dye solution. The dye solution and adsorbent mixture were shaken by a water bath shaker for 24 h at 120 rpm at 25 °C. Upon reaching equilibrium, the residual concentrations of methylene dye, crystal violet dye, Coomassie brilliant blue dye and naphthol black dye in the solution phase were determined using a UV–Vis spectrophotometer at 664, 589, 555 and 616 nm, respectively, with the aid of a calibration curve plotted between the concentration of known solutions and their corresponding absorbance values.

The effect of pH on the adsorption of CV dye using PPAC and S-PPAC was investigated for pH values ranging from 3 to 10. The contact time effect was studied for contact times ranging from 5 to 920 min. The effect of the initial CV concentration on adsorption was measured at temperatures ranging from 25 to 40 °C. The following parameters were maintained constant: adsorbent dosage (5 mg), pH (10), agitation speed (120 rpm) and contact time (24 h).

To establish the zero-point charge (pH_ZPC_), a 10 mL solution of 0.1 M KCl was prepared, and the pH was varied between 2 and 11 by adding either 0.1 M HCl or 0.1 M NaOH solution. Following that, 10 mg of each sample was added to the solution and shaken for 24 h. Then, the final pH of the solution was determined.

The statistical analysis and equations of the isotherm and kinetic models used in this study are summarized in the Supporting Information.

## 4. Conclusions

In conclusion, we successfully prepared sulfonated activated carbon (S-PPAC) as an effective adsorbent for cationic dyes removal. The activated carbon (PPAC) was prepared by single-step carbonization/activation from pomegranate peel waste. Microscopic and spectroscopic analysis confirmed the successful preparation of sulfonated S-PPAC, where the 5-sulfonate-salicylaldehyde sodium salt agent effectively attached to the carbon atoms of PPAC, and the surface area of the PPAC decreased from 1180.63 to 740.75 m^2^/g after the surface modification. To compare the adsorption capacities of PPAC and S-PPAC to remove various types of organic dyes, a comparative analysis was performed. The results show that the crystal violet dye had the maximum adsorption capacity on both adsorbents. The S-PPAC showed significantly enhanced adsorption capacity compared to the unmodified PPAC. At temperatures ranging from 25 to 45 °C, the S-PPAC achieved adsorption capacities of 667.84, 689.75, 731.59, 764.22 and 785.53 mg/g, while the PPAC reached capacities of 552.79, 570.79, 584.36, 600.1 and 606.7 mg/g, respectively. Under optimal conditions, the adsorption capacity of S-PPAC was enhanced by 20% due to the presence of sulfonate groups on its surface. The isotherm results show that the Freundlich model was the best fit for describing adsorption, whereas the Elovich model was the best fit to describe the adsorption kinetics. The thermodynamic experiments proved the spontaneous, favorable and endothermic nature of CV dye adsorption onto PPAC and S-PPAC. Finally, the S-PPAC can be reused up to three times without any loss in adsorption efficiency. This finding highlights the potential of S-PPAC as a promising adsorbent for removing dyes from real polluted water.

## Figures and Tables

**Table 1:** Textural properties of the PPAC and S-PPAC samples.

Sample	S_BET_ (m^2^/g)	S_micro_	V_T_ cm^3^/g	V_micro_ cm^3^/g	V_non-micro_ cm^3^/g	V_micro_/V_T_ (%)	D_p_ (nm)	APS (nm)
PPAC	1180.63	890.74	0.6644	0.4706	0.1938	70.83	2.25	5.08
S-PPAC	740.75	555.84	0.4210	0.2929	0.1281	69.57	2.27	8.10

Note: S_BET_: BET surface area; V_T_: total pore volume; S_Micro_: micropore surface area; V_Micro_: micropore volume; D_p_: pore width; APS: average pore size.

**Table 2:** Isotherm parameters for adsorption of CV dye onto PPAC and S-PPAC at different temperatures.

Model	PPAC					S-PPAC				
	298 K	303 K	308 K	313 K	318 K	298 K	303 K	308 K	313 K	318 K
q_e,exp_	578	604	626	640	650	706	720	754	790	810
Langmuir										
q_max_ (mg/g)	552.79	570.79	584.36	600.1	606.7	667.84	689.75	731.59	764.22	785.53
K_L_ (L/mg)	0.4432	0.5081	1.36	1.25	1.21	1.37	1.24	0.5026	0.4357	0.3980
R^2^	0.8786	0.8815	0.8816	0.8845	0.8854	0.9181	0.9230	0.9328	0.9350	0.9362
SE	41.08	41.88	42.22	43.01	43.45	40.89	41.35	42.72	44.49	45.78
Freundlich										
K_F_ (mg/g)/(mg/L)^n^	325.87	328.37	361.49	359.81	358.42	412.94	420.40	399.32	394.16	391.52
1/n	0.093	0.098	0.087	0.094	0.097	0.090	0.092	0.11	0.121	0.128
R^2^	0.8923	0.8990	0.9028	0.9075	0.9094	0.9299	0.9334	0.9372	0.9428	0.9456
SE	87.76	85.15	79.84	79.71	79.56	68.56	67.62	69.66	68.33	67.84
Temkin										
β (J/mol)	43.25	46.24	40.98	44.61	46.21	49.95	53.29	66.57	75.22	80.85
AT (L/mg)	1229.2	844.72	6571.13	3039.90	2227.0	3169.4	2096.8	253.97	115.44	75.91
b_T_ = RT/β	57.28	54.47	62.48	58.33	57.21	49.60	47.27	38.47	34.60	32.70
R^2^	0.8914	0.8975	0.9004	0.9050	0.9066	0.9220	0.9265	0.9318	0.9383	0.9416

**Table 3:** Comparison of q_max_ for PPAC and S-PPAC with some reported activated carbon-based adsorbents.

Adsorbent	Source of AC	Activation	BET (m^2^/g)	q_max_ (mg/g)	Ref.
AC/Fe_3_O_4_	Lemon wood	pyrolysis	38.69	35.3	[37]
AC	Rice husk	H_2_SO_4_	681	64.87	[40]
CoFe_2_O_4_/AC	Commercial	-	109.9	184.2	[39]
AC	Date palm petioles	pyrolysis	640	209	[34]
Ag NPs/AC	Commercial	pyrolysis	-	87.2	[41]
AC	Coconut husk	H_2_SO_4_	-	418	[42]
AC-Fe_3_O_4_-Chitosan	Commercial	HNO_3_	-	505.87	[43]
AC	P(1,5-DANPh)	KOH	1679	487.53	[44]
AC	Poultry litter	ZnCl_2_	148.05	70.32	[45]
AC	Moringa oleifera	H_3_PO_4_	1394	469.55	[46]
AC	Ferula orientalis	ZnCl_2_	1476	769.23	[47]
AC	Chitin	KOH	2186.3	420	[38]
PPAC	Pomegranate peel waste	KOH	1180.63	606.7	This study
S-PPAC			740.75	785.53	

**Table 4:** Kinetic parameters values for PFO, PSO and Elovich models for adsorption of CV dye onto PPAC and S-PPAC.

	Adsorbent	
Applied Model	PPAC	S-PPAC
q_t,exep_	204	242
PFO		
q_e,cal_	182.59 ± 4.68	222.48 ± 3.39
k_1_	0.0447 ± 0.0094	0.0575 ± 0.0094
R^2^	0.99691	0.99818
χ^2^	228.81801	133.62
PSO		
q_t, cal_	194.37 ± 4.28	232.12 ± 2.63
K_2_	0.00037 ± 0.00008	0.00054 ± 0.00008
R^2^	0.99858	0.99938
χ^2^	105.40	45.36
Elovich		
α	713.24 ± 238.51	42,698.53 ± 10,238.40
β	0.05055 ± 0.00219	0.06015 ± 0.00122
R^2^	0.99978	0.99996
χ^2^	16.27	2.66
Intraparticle diffusion		
K_ip(1)_	8.37	6.25
C_1_	92.70	149.41
K_ip(2)_	3.68	2.35
C_2_	120.36	185.40
K_ip(3)_	0.8250	0.45558
C_3_	180.16	226.25

**Table 5:** Values of thermodynamic parameters for the adsorption of CV dye onto PPAC and S-PPAC.

Adsorbent	Temperature (K)	Van’t Hoff Equation	K_C_	ΔG^ο^ (KJ/mol)	ΔH^ο^ (KJ/mol)	ΔS^ο^ (J/mol K)
PPAC	298	y = −872.32x + 4.77 R^2^ = 0.9822	6.33333	−4.57	7.25	39.66
	303		6.57143	−4.74		
	308		6.95522	−4.97		
	313		7.375	−5.20		
	318		7.52381	−5.34		
S-PPAC	298	y = −2526.12x + 9.78 R^2^ = 0.9933	3.71429	−3.25	21.00	81.28
	303		4.13497	−3.56		
	308		4.80272	−4.02		
	313		5.63359	−4.50		
	318		6.19672	−4.82		

## Data Availability

Data are contained within the article and Supplementary Materials.

## Supplementary Materials

The following supporting information can be downloaded at: https://www.mdpi.com/article/10.3390/molecules28237712/s1, The statistical analysis and equations of the isotherm and kinetic models used in this study are summarized in the supporting information. Refs. [50,51,52,53,54,55] are cited in supplementary file.
