# Supplementary material for: Enhanced Adsorption of Textile Dyes by a Novel Sulfonated Activated Carbon Derived from Pomegranate Peel Waste: Isotherm, Kinetic and Thermodynamic Study
Source: Molecules. 2023 Nov 22;28(23):7712. doi: 10.3390/molecules28237712 (PMC10708109; doi:10.3390/molecules28237712)
Supplement: Supplementary file 1 [file molecules-28-07712-s001.zip › molecules-2712155-supplementary.pdf]

Enhanced adsorption of textile dyes by a novel sulfonated activated carbon derived from pomegranate peels waste: Isotherm, kinetic and thermodynamic study

Badr M. Thamer[1*], Faiz A. Al-aizari and Hany S. Abdo[2]

[1]Department of Chemistry, College of Science, King Saud University, P.O. Box 2455, Riyadh 11451, Saudi Arabia

[2]Mechanical Engineering Department, College of Engineering, King Saud University, Riyadh, 11421, Saudi Arabia

*Corresponding author; bthamer@ksu.edu.sa

S1. Calculation of surface rea

The textural properties were measured by collecting nitrogen adsorption–desorption isotherms (Micromeritics ASAP 2020 sorptometer) at 77 K. The Brunauer– Emmett–Teller (BET) method was applied to calculate the specific surface area (SBET; m^2/g). The microporous surface area ratio was calculated through the following equation:

$$\%\,microporous\ area\ = \frac{S_{Micro}}{S_{BET}}\ x\ 100$$

S_{micro} and S_{BET} are microporous surface area and BET surface area, respectively.

S2. Statistical analysis

Recently, nonlinear regression method is one of the most reliable tools to define the best fitting experimental data of adsorption and kinetic process. To determine the best kinetic and isotherm model for representing experimental data, Chi-square values (χ^2) and coefficient of determination values (R^2) were calculated for the nonlinear method by Eq.S4 and Eq.S6, respectively. The model with the lowest value of χ^2 and the highest value of R^2 is the most representative of the experimental data and the least error.

$$\chi^2 = \sum_i^n \frac{(q_{e,exp} - q_{e,model})^2}{q_{exp}} \quad (S1)$$

$$R^2 = 1 - \frac{\sum(q_{e,exp} - q_{e,model})^2}{\sum(q_{e,exp} - q_{e,mean})^2} \quad (S2)$$

where ($q_{e,model}$) is each value of q_e predicted by the fitted model, ($q_{e,\,exp}$) is each value of q_e measured experimentally, ($q_{e,mean}$) is the average value of q_e that was measured experimentally, n_p is the number of performed experiments, and p is the number of parameters for the fitted model.

S3. Calculation of adsorption capacity

Equations 1-3s were applied to measure the adsorption capacity at specific time t (qt) and equilibrium (q_e) and separation efficiency.

$$q_t = \frac{C_o - C_t}{m} \times V \quad (S3)$$

$$q_e = \frac{C_o - C_e}{m} \times V \quad (S4)$$

$$Removal\ \ effeciency\,(\%) = \frac{C_o - C_e}{C_o} \times 100 \quad (S5)$$

C_o (mg/L) is the initial concentration, C_t (mg/L) is the concentration at a specific time, and C_e (mg/L) is the concentration of dye at the equilibrium state. V (L) refers to the used volume of dye solution, and the mass of adsorbents was denoted by m (g).

S4. Adsorption isotherm models

S4.1. Langmuir isotherm

The Langmuir isotherm model assumes homogeneity of the adsorbent surface and monolayer coverage by the adsorbate. This model can be represented by the following non-linear equation [1]:

$$q_e = \frac{Q_o K_L C_e}{1 + K_L C_e} \quad (S6)$$

Where q_e is the amount of adsorbate (mg/g), C_e is the equilibrium concentration of adsorbate in solution (mg L^{-1}), K_L is the Langmuir constant (L mg^{-1}), and Q_o (mg/g) is the monolayer adsorption capacity.

S4.2. Freundlich isotherm

The Freundlich isotherm model more accurately describes heterogeneous surfaces or multilayers of adsorbent and can be described as follows [2]:

$$q_e = K_f \, C_e^{1/n} \qquad \text{(S7)}$$

K_f is the Freundlich constant related to the adsorption capacity, and *1/n* is the constant related to adsorption strength. If the value of *1/n* is between 0.1 and 1.0, the adsorption is favorable.

S4.3. Temkin isotherm

Temkin isotherm is the early model describing the adsorption of hydrogen onto platinum electrodes within the acidic solutions. This isotherm contains a factor that explicitly taking into the account of adsorbent–adsorbate interactions. It can be describes as follows [3]:

$$q_e = B_t \ln(A_t C_e) \qquad \text{(S8)}$$

Where A_t is the Temkin equilibrium constant (L g^{-1}) corresponding to the maximum binding energy, B_t = RT/b and b is related to heat of adsorption (J mol^{-1}).

S5. Kinetic Studies of Adsorption

The adsorption rate is another important factor to optimum operating condition as it provides valuable information about the reaction pathways and the mechanism of adsorption reactions. There are many kinetic models which have been developed for the determination of adsorption rate. The most commonly used are Pseudo-first-order model (PFO), Pseudo-second-order model (PSO), Elovich model and intra-particle diffusion model.

S5.1 Pseudo-first-order model (PFO)

PFO model is a nonlinear relationship and it can be represented as follow [4]:

$$q_t = q_e(1 - e^{-K_1 t}) \qquad \text{(S9)}$$

In which, q_t and q_e are the amounts of adsorbate adsorbed at time *t* and equilibrium, respectively, K_1 (min^{-1}) is the rate constant of the PFO.

S5.2 Pseudo-second-order model (PSO)

PSO can be inferred from this model that the chemisorption is vital in the rate determining step (Ho and McKay, 1999). The nonlinear form of pseudo-second-order model can be expressed as [5]:

$$q_t = \frac{q_2^2 k_2 t}{1 + q_e K_2\ t} \qquad \text{(S10)}$$

Where K_2 (g mg^{-1} min^{-1}) is the rate constant of the PSO adsorption process.

S5.3 Elovich kinetic model

The Elovich kinetic model is another model that can be applied for the study of the chemisorption. In the absence of desorption, the reaction rate will decrease owing to the increasing surface coverage. The equation can be shown as [6]:

$$q_t = \frac{1}{\beta} \ln(1 + \alpha\beta t) \qquad \text{(S11)}$$

Where α (mg/g min^{-1}) is the initial adsorption rate; β (mg/g) is desorption constant during any one experiment.

References:

[1] I. Langmuir, The adsorption of gases on plane surfaces of glass, mica and platinum, J. Am. Chem. Soc. 40 (1918) 1361–1403. https://doi.org/10.1021/ja02242a004.

[2] H.M.F. Freundlich, Over the adsorption in solution, Z. Phys. Chem. 57 (1906) 385–471.

[3] and V.P. Tempkin, M. I., Kinetics of ammonia synthesis on promoted iron catalys, Acta Phys. Chim USSR. 12 (1940) 327.

[4] S. Lagergren, About the theory of so-called adsorption of soluble substances, K. Sven. Vetenskapsakademiens Handl. 24 (1898) 1–39.

[5] Y.S. Ho, D.A.J. Wase, C.F. Forster, Kinetic Studies of Competitive Heavy Metal Adsorption by Sphagnum Moss Peat, Environ. Technol. 17 (1996) 71–77. https://doi.org/10.1080/09593331708616362.

[6] S.H. Chien, W.R. Clayton, Application of Elovich Equation to the Kinetics of Phosphate Release and Sorption in Soils1, Soil Sci. Soc. Am. J. 44 (1980) 265. https://doi.org/10.2136/sssaj1980.03615995004400020013x.
